# Impact of Single Amino Acid Substitutions in Parkinsonism-Associated Deglycase-PARK7 and Their Association with Parkinson’s Disease

**DOI:** 10.3390/jpm12020220

**Published:** 2022-02-05

**Authors:** Farah Anjum, Namrata Joshia, Taj Mohammad, Alaa Shafie, Fahad A. Alhumaydhi, Mohammad A. Aljasir, Moyad J. S. Shahwan, Bekhzod Abdullaev, Mohd Adnan, Abdelbaset Mohamed Elasbali, Visweswara Rao Pasupuleti, Md Imtaiyaz Hassan

**Affiliations:** 1 Department of Clinical Laboratory Sciences, College of Applied Medical Sciences, Taif University, P.O. Box 11099, Taif 21944, Saudi Arabia; f2016anjum@gmail.com (F.A.); dr.alaa.shafie.tu@gmail.com (A.S.); 2 Department of Computer Science, Jamia Millia Islamia, Jamia Nagar, New Delhi 110025, India; namrata8750451546@gmail.com; 3 Centre for Interdisciplinary Research in Basic Sciences, Jamia Millia Islamia, Jamia Nagar, New Delhi 110025, India; taj144796@st.jmi.ac.in; 4 Department of Medical Laboratories, College of Applied Medical Sciences, Qassim University, Buraydah 52571, Saudi Arabia; f.alhumaydhi@qu.edu.sa (F.A.A.); Mjasr@qu.edu.sa (M.A.A.); 5 College of Pharmacy & Health Sciences, Ajman University, Ajman 20550, United Arab Emirates; m.shahwan@ajman.ac.ae; 6 Scientific Department, Akfa University, Tashkent 100095, Uzbekistan; b.abdullaev@akfauniversity.org; 7 Department of Biology, College of Science, University of Hail, Hail 55436, Saudi Arabia; drmohdadnan@gmail.com; 8 Clinical Laboratory Science, College of Applied Sciences-Qurayyat, Jouf University, Sakaka 72388, Saudi Arabia; aeelasbali@ju.edu.sa; 9 Department of Biomedical Sciences and Therapeutics, Faculty of Medicine & Health Sciences, Universiti Malaysia Sabah, Kota Kinabalu, Sabah 44800, Malaysia; 10 Department of Biochemistry, Faculty of Medicine and Health Sciences, Abdurrab University, Pekanbaru, Riau 28291, Indonesia; 11 Centre for International Collaboration and Research, Reva University, Rukmini Knowledge Park, Katti-genahalli, Yelahanka, Bangalore, Karnataka 560064, India

**Keywords:** Parkinson’s disease protein 7, protein deglycase DJ-1, Parkinson’s disease, single amino acid substitution, structural dysfunctions in PARK7/DJ-1

## Abstract

Parkinsonism-associated deglycase-PARK7/DJ-1 (PARK7) is a multifunctional protein having significant roles in inflammatory and immune disorders and cell protection against oxidative stress. Mutations in *PARK7* may result in the onset and progression of a few neurodegenerative disorders such as Parkinson’s disease. This study has analyzed the non-synonymous single nucleotide polymorphisms (nsSNPs) resulting in single amino acid substitutions in *PARK7* to explore its disease-causing variants and their structural dysfunctions. Initially, we retrieved the mutational dataset of *PARK7* from the Ensembl database and performed detailed analyses using sequence-based and structure-based approaches. The pathogenicity of the *PARK7* was then performed to distinguish the destabilizing/deleterious variants. Aggregation propensity, noncovalent interactions, packing density, and solvent accessible surface area analyses were carried out on the selected pathogenic mutations. The SODA study suggested that mutations in *PARK7* result in aggregation, inducing disordered helix and altering the strand propensity. The effect of mutations alters the number of hydrogen bonds and hydrophobic interactions in PARK7, as calculated from the Arpeggio server. The study indicated that the alteration in the hydrophobic contacts and frustration of the protein could alter the stability of the missense variants of the PARK7, which might result in disease progression. This study provides a detailed understanding of the destabilizing effects of single amino acid substitutions in PARK7.

## 1. Introduction

Parkinsonism-associated deglycase-PARK7/DJ-1 (PARK7) is a multifunctional protein that plays a crucial role in inflammatory diseases, immune disorders, and cell protection against oxidative stress. It is localized in the nucleus, cytoplasm and mitochondria [1]. It belongs to the superfamily PfpI/Hsp31/DJ-1 with a conserved exposed cysteine residue [2]. It has various functions such as a peroxidase, a protease, glyoxalase, a chaperone for synuclein, and an apoptosis inhibitor [2]. It prevents the cell from oxidative stress, associated with various complex disorders such as Parkinson’s disease, cancer, asthenozoospermia, and Alzheimer’s disease [3,4]. Parkinson’s disease is an untreatable, unstoppable growing neurodegenerative disorder, positioned as the second most common neurodegenerative disorder after Alzheimer’s disorder [5]. It is progressively noticeable as a multicentred disorder that affects the nervous system and results in rest tremor, postural tremor, muscle stiffness, and bradykinesia [5].

Apart from Parkinson’s disease, *PARK7* has been involved in various complexities such as cancer and infertility [6,7]. A study in the Netherlands and Italy found that families have early-onset autosomal recessive Parkinson’s disease due to the homozygous mutations in the *PARK7* gene [8]. *PARK7* is found in the cytoplasm and nucleus, whose expression has been seen in many tissues, including brain, eye, and endocrine tissues [8]. In the subcortical region, high expression of *PARK7* mRNA may be crucial for basal ganglia function [8]. On overexpression models, the subcellular distribution shows that *PARK7* is mainly traced in the nucleus and the cytoplasm, whereas its lesser amount exists in mitochondria under stress conditions [6]. In the mouse brain, the endogenous poll of *PARK7* in the inter-membrane space and mitochondrial matrix was also shown by subcellular fractionation and immunogold electron microscopy [6].

In humans, the *PARK7* gene is found at chromosome 1p36, which encodes a 189 amino acid residue long protein that shows structural similarity to the THiJ and PfpI bacterial proteins involved in protease activity and thiamine synthesis, respectively [8]. The *PARK7* structure has been analyzed in detail, and the crystal structure of both the monomer and dimer was reported by an independent report [6]. *PARK7* as a monomer comprises 8 alpha-helix, and 11 beta-strands ordered asymmetrically in a helix–strand–helix sandwich similar to the Rossman fold [9]. Eight pairs of hydrogen bonds and various van der Waal interactions form dimerization of *PARK7* [6,9]. Proteolytic activity is displayed at a conserved cysteine residue by its homolog PH1704. C106, a highly conserved cysteine residue of PARK7, has a possible function as a protease. The main catalytic triad of Cys–His–Glu in the PH1704 active site is absent in PARK7, along with C106.

*PARK7* loses its functionality when it becomes mutated, and it is related to mitochondria dysfunction, resulting in the early onset of Parkinson’s disease [10,11]. There is inadequacy in sperm motility in humans and other species, and infertility was also observed due to the *PARK7* mutations [10]. Mutation-associated loss of function in *PARK7* is related to the mitochondria’s abnormal morphology and dynamics, alteration in calcium homeostasis, and increased sensitivity to oxidative stress [10]. *PARK7* was recognized as a part of the novel glyoxalase family examined as detoxifying proteins [10]. *PARK7* acts as both a causative and carcinogenic gene and plays a crucial role in oxidative stress [10]. Because mutations in *PARK7* cause various complex diseases, we studied several variants of *PARK7* using state-of-the-art computational approaches [12,13,14,15,16]. We took 152 mutations of the whole protein to explore their consequences in disease progression. The present study will offer an in-depth analysis of mutations on the structure of *PARK7* and their possible implication in Parkinson’s disease.

## 2. Methods and Materials

### 2.1. Data Resources and Tools

**Figure 1:**
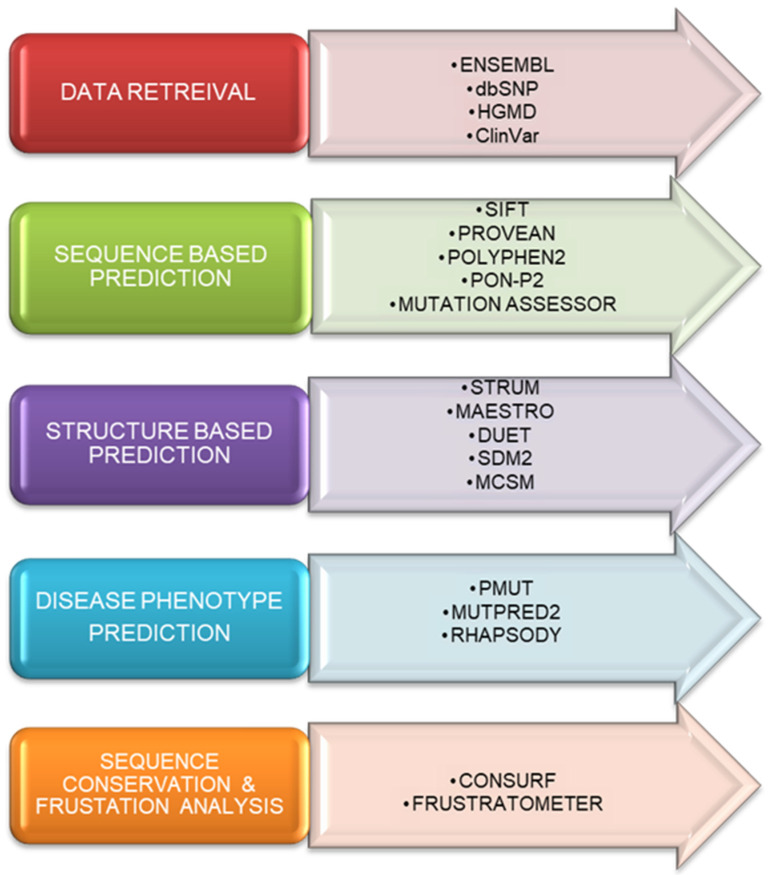
Overview of the computational aspects to predict the pathogenic mutations of the *PARK7* protein at the sequential, structural, and functional levels.

The protein sequence of *PARK7* was downloaded from the UniProt database (ID: Q99497). A list of nonsynonymous single nucleotide polymorphisms (nsSNPs) was prepared from the Ensembl [17], dbSNP [18], HGMD [19], and ClinVar [20] databases. The duplicate variants were removed from the list. The *PARK7* protein structure was downloaded from the RCSB Protein Data Bank (PDB ID: 1P5F). We used multiple tools for sequence-based and structure-based predictions to enhance the confidence score of the predicted results [21,22,23,24]. The overview of the computational aspects to predict the pathogenic mutations in *PARK7* is illustrated in Figure 1.

### 2.2. Sequence-Based Prediction

#### 2.2.1. SIFT

The SIFT (http://sift.jcvi.org/, accessed on 1 December 2021) tool is used to examine whether a mutation in a protein is deleterious or not based on the physical characteristics of the amino acid. It also considers the sequence homology of a protein. If the SIFT score is less than or equal to 0.05, then the mutation is predicted as deleterious. The SIFT tool predicts the effect of these missense variants on the protein. A total of 152 missense variants were examined for *PARK7* and categorized as deleterious/neutral or with unknown significance.

#### 2.2.2. PolyPhen-2

PolyPhen-2 (http://genetics.bwh.harvard.edu/pph2/, accessed on 1 December 2021) is another sequence-based tool that predicts the damaging probability of mutation by considering the physical and comparative properties of the sequences. It provides the PSIC (Position-Specific Independent Count) score for the missense variants and then calculates the score divergence with the wild-type.

#### 2.2.3. PROVEAN

PROVEAN (http://provean.jcvi.org/, accessed on 1 December 2021) also predicts the deleterious impact in a protein. Here, if the calculated score is less than −2.5 for a mutation, it is considered damaging, whereas a mutation with scores greater than −2.5 is considered neutral.

#### 2.2.4. Mutation Assessor

Mutation Assessor (http://mutationassessor.org/r3/, accessed on 1 December 2021) is another sequence-based tool used to analyze the effect of missense mutations in a protein. It is based on evolutionarily conserved residues and a multiple sequence alignment approach. This tool accepts UniProt accession ID as input for protein sequence. It categories the missense mutations as neutral, low, or medium for damaging effects. It provides an FI score for each mutation. If the FI score is greater than 2.00, the missense mutation is considered damaging.

#### 2.2.5. PON-P2

PON-P2 (http://structure.bmc.lu.se/PON-P2/, accessed on 1 December 2021) is another sequence-based approach that predicts pathogenic missense mutations using a machine learning technique. This tool categorizes the missense variants of a protein into unknown, neutral, and pathogenic categories. It gives results in less amount of time. It uses physical properties, evolutionary sequence conservation, and biochemical properties of the protein. The missense variants data to PON-P2 can be submitted in different file formats.

### 2.3. Structure-Based Prediction

#### 2.3.1. SDM2

SDM2 (http://marid.bioc.cam.ac.uk/sdm2, accessed on 1 December 2021) is a structure-based tool that estimates the change in protein stability between the wild-type and the mutant. It accepts the PDB file format as input. The SDM2 server predicts the OSP (residue-occluded packing density), RSA (relative side-chain solvent accessibility), and residue depth for the mutant and wild-type protein. As a score, if the ΔΔ*G* is >0 for a given mutant, SDM2 predicts it destabilizing.

#### 2.3.2. MCSM

mCSM (http://biosig.unimelb.edu.au/mcsm/, accessed on 1 December 2021) is a web server used to predict destabilizing mutations of a protein using a graph-based approach. This tool provides better insights into the missense mutations associated with different disorders. A missense mutation is considered as destabilizing if the mCSM score (ΔΔ*G*) < 0.

### 2.4. Identification of Pathogenic nsSNPs

#### 2.4.1. PhD-SNP

PhD-SNP (http://gpcr.biocomp.unibo.it/cgi/predictors/PhD-SNP/PhD-SNP.cgi/, accessed on 1 December 2021) is a structure-based tool that differentiates disease-causing nsSNPs from neutral. This is an SVM-based tool that depends on the local environment of substitution for prediction. PhD-SNP accepts FASTA file format or PDB ID of the protein as an input. This tool classifies mutation into disease or neutral. Its prediction is based on sequence-based, profile-based, and hybrid methods.

#### 2.4.2. Rhapsody

Rhapsody (http://rhapsody.csb.pitt.edu/, accessed on 1 December 2021) is a web server that predicts the mutant protein’s sequence conservation and structural properties. Rhapsody predicts residue-averaged pathogenicities of the missense mutations better than EVmutation and PolyPhen-2. Rhapsody accepts the PDB ID of a protein as an input. This tool can submit the batch query (up to 10,000) of missense variants, i.e., less time-consuming.

### 2.5. Analysis of Packing Density

The replacement of a large amino acid with a smaller one can alter residue depth and solvent accessibility and influence the formation of active-site cavities in proteins [25]. Apart from the studies mentioned above, we calculated the OSP, RSA, and residue depth for the mutant and wild-type protein using the SDM2 web-based server. SDM2 is a freely accessible and user-friendly interface for studying proteins. It uses an environment-specific missense mutation table to evaluate RSA, OSP, and residue depth. RSA value is calculated using Lee and Richard’s method. RSA, residue depth, and OSP are considered important properties of the protein structure to predict the stability of the protein.

### 2.6. Analysis of Aggregation Propensity

SODA (http://protein.bio.unipd.it/soda/, accessed on 1 December 2021) is a sequence- and structure-based approach for the prediction of the solubility of a protein. This tool estimates the protein’s aggregation, disorder, helix, and strand propensity, which rise due to the missense mutations. SODA accepts both FASTA as well as PDB file format for input.

### 2.7. Analysis of Noncovalent Interactions

Arpeggio server (http://biosig.unimelb.edu.au/arpeggioweb/, accessed on 1 December 2021) is a web-based tool that calculates the number of interatomic interactions like van der Waal interactions, hydrogen bonds, aromatic interactions, and hydrophobic interactions of a protein structure. Arpeggio can estimate about 15 types of interatomic interactions. This tool accepts PDB file format as input. It provides a downloadable list of the number of different types of interactions.

### 2.8. Conservation Analysis

Conservation plays a vibrant role in the structure and function of any protein. Conservation analyses can be performed using multiple sequence alignments of similar proteins. A web tool named ConSurf (https://consurf.tau.ac.il/, accessed on 1 December 2021) measures the degree of conservation of the sequence. We have used ConSurf-DB, which has pre-calculated evolutionary profiles of various proteins with known structures.

### 2.9. Frustration Analysis

The Frustratometer web tool (http://frustratometer.qb.fcen.uba.ar/, accessed on 1 December 2021) was used to examine the residual frustration in *PARK7* and its mutants. It evaluates the energy of a protein structure and compares it to the energy of a set of ‘decoy’ states. The single and configurational residual indexes for all three systems were computed. In this calculation, contact is highly frustrated/destabilizing if the value of the Z-score is <0.78. In contrast, contact is minimally frustrated/stabilizing if the value of the Z-score is >0.78. At the same time, if the energy lies in between these two values, the contact is considered neutral.

## 3. Result and Discussion

**Figure 2:**
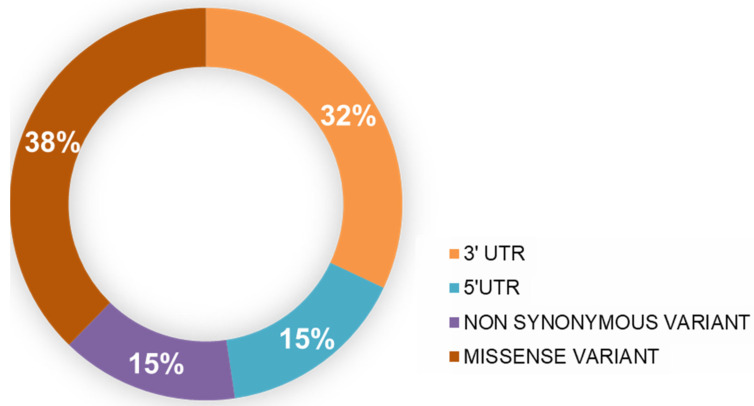
Representation of four different types of SNPs in *PARK7* using the Ensembl genome browser.

At the beginning of the study, a total of 152 reported mutations were retrieved from the Ensembl (https://asia.ensembl.org/index.html, accessed on 1 December 2021) database. This study is based on the sequence- and structure-based analyses of these mutations retrieved from the Ensembl. A multilevel approach was operated to predict the functional and structural effect of mutations on the *PARK7* protein. Sequence-based and structural-based approaches have been operated to obtain the high confidence diseased mutations [26,27,28]. All of the mutations were sequence-based analyzed using five web servers, which were PON-2, Mutation Assessor, SIFT, PolyPhen2, and PROVEAN. Only the high confidence mutations (predicted to be ‘deleterious’ by at least three predictors) of the *PARK7* protein were taken in the structure-based analysis. Then, the stability of the selected mutations was predicted using SDM2, mCSM, and MUpro. The input file format in all of these structure-based stability prediction tools was the PBD coordinates file of the protein. For further study, the mutations whose stability decreased were selected for the disease phenotype analysis using Rhapsody, PMut, PhD-SNP, and MutPred2. The packing density and accessible surface area, degree of solubility, and aggregation propensity were also studied using different approaches. The *PARK7* nsSNPs retrieved from the Ensembl database were categorized into four types, graphed in Figure 2.

### 3.1. Sequence- and Structure-Based Identification of Diseased Mutations

**Figure 3:**
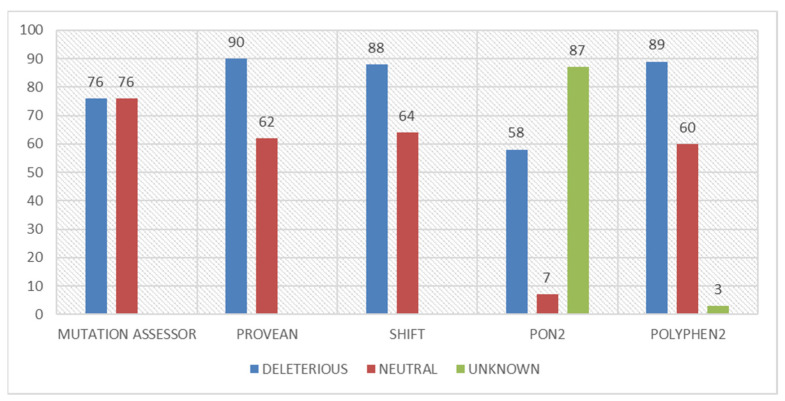
Deleterious, unknown, and neutral missense mutations’ distribution identified by sequence-based approaches for the entire sequence of the *PARK7* protein.

Multiple tools based on different algorithms and approaches were used to identify diseased mutations since using only one tool can provide some false positives. Multiple tools were used to avoid any false prediction, warranting more accuracy of the outcomes. For the sequence-based analysis, PON-2, Mutation Assessor, SIFT, PolyPhen2, and PROVEAN were used. All 152 mutations of the human *PARK7* were firstly analyzed using sequence-based tools (Table S1). PolyPhen2, PON-2, SIFT, PROVEAN, and Mutation Assessor inferred that out of the 152 mutations, 89 (58.55%), 58 (38.15%), 88 (57.89%), 90 (59.215), and 76 (50%) were deleterious, respectively (Figure 3).

**Figure 4:**
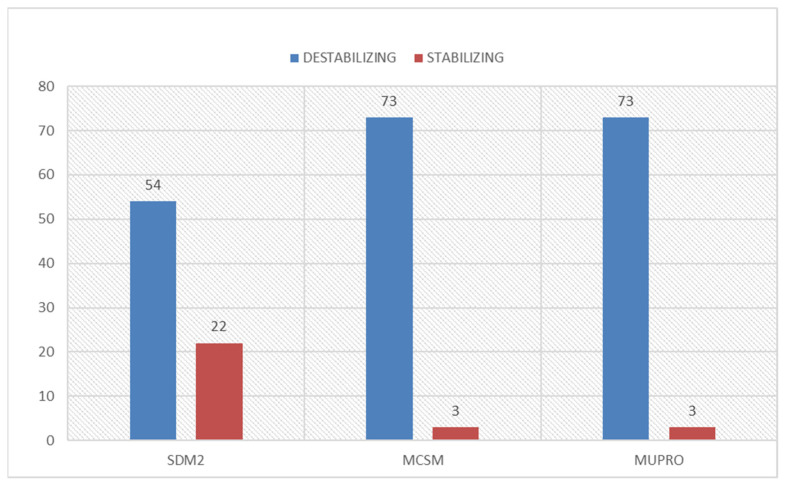
Destabilizing and stabilizing missense variants’ distribution identified by structure-based approaches for high confidence mutations of *PARK7* protein.

From the above-mentioned sequence-based analysis output, structure-based stability prediction using SDM2, MUpro, and mCSM was carried out. The structure-based approach was performed on the 76 variants with high confidence deleteriousness from the sequence-based predictions (Table S2). Out of the 76 variants, SDM2, mCSM, and MUpro predicted 54 (71.05%), 73 (96.05%) and 73 (96.05%) missense variants as destabilizing, respectively (Figure 4). Furthermore, to raise the confidence level, we selected those variants identified as diseased by at least three different sequence-based approaches and at least two different structure-based approaches. After investigating by using this approach, 69 (45.39% of the total) variants were selected, which were identified as diseased/deleterious or destabilizing by using both approaches. The disease phenotype identification was carried out on these 69 missense variants.

### 3.2. Disease Phenotype Identification of Missense Variants

We identified the disease phenotype associated with the selected mutations using the Rhapsody and PhD-SNP web servers. Based on the pathogenicity score obtained by these web servers, we predicted the disease phenotype of the selected variants (Table 1). The Rhapsody and PhD-SNP web servers categorized the missense variants into ‘neutral’ or ‘diseased/deleterious’ mutations. From the 69 missense variants, we identified that 25 mutations were predicted to be diseased in both predictors.

### 3.3. Packing Density and Accessible Surface Area Analysis

Change in solvent accessibility is considered one of the critical parameters to understand the structural features of proteins [25]. Any replacement of a large amino acid with a smaller one can alter residue depth and solvent accessibility and influence the formation of cavities [29]. Analysis of solvent accessibility provides information about the packing density before and after achieving mutation. Using the SDM2 server, we calculated the OSP, RSA, and residue depth for 25 mutants of *PARK7* and its wild-type structure (Table 2). The change in OSP, RSA, and residue depth of the selected variants resulted in the identification of 21 mutations that reduce the structural stability and integrity of the *PARK7* protein.

### 3.4. Aggregation Propensity Analysis

The solubility of a protein influences its function to a great extent. Diseases such as Parkinson’s disease, amyloidosis, and Alzheimer’s disease are caused by the aggregation of insoluble parts of the proteins [30,31,32,33,34]. To predict the solubility of PARK7, we calculated the variants’ solubility using SODA (Solubility based on Disorder and Aggregation). Mutations alter the aggregation, disorder, helix, and strand propensity of the protein variants, and these parameters were predicted by SODA. From the disease phenotype prediction of 21 high confidence missense variants, 12 variants raised the solubility of the *PARK7* protein, whereas the rest decreased the solubility of the protein (Table 3).

### 3.5. Noncovalent Interaction Analysis

It is known that the alteration in the hydrophobic contacts of a protein can alter its stability [35]. Missense mutations in *PARK7* protein can induce large alterations; thus, they can affect the structural stability of the protein. Using the Arpeggio server, we calculated the number of hydrogen bonds, ionic interactions, van der Waal interactions, electrostatic, and hydrophobic interactions of the *PARK7* mutants (Table 4). The effect of mutation is shown by decreasing and increasing the number of different types of bonds. All selected 21 missense variants were examined with the help of the Arpeggio server. We found that *PARK7* loses several contacts, especially hydrophobic and van der Waals interactions, after obtaining most of the selected mutations compared to the wild-type protein (Table 4).

Parkinson’s disease is specified by dopaminergic dysfunction. Mutation in *PARK7* has been related to early-onset Parkinson’s disease [6,36]. After an extensive literature survey, we discovered that out of 21 mutations, there are 8 pathogenic mutations (L10P, G13E, E16G, A104T, A107P, T154A, L166P, and L172Q) that have been widely explored in various research works. Here, two mutations (L166P and L172Q) are damaging, but their structural consequences are not studied much. L172Q mutation resulting from an SNP does not reduce the expression of *PARK7* mRNA but somehow destabilizes the protein to a point where it is barely detectable by western blot [37,38]. L172Q is highly unstable, rapidly degraded by the proteasome, behaves very similar to L166P, and possibly retains chaperoning activity [6,39]. Studies reported that wild-type and missense mutants (i.e., M26I, R98Q, A104T, and D149A) of *PARK7* were found to be stable proteins, whereas only the L166P mutation was unstable in cells [6,39,40,41]. At the same time, the L166P mutant was degraded by proteasome-mediated endoproteolytic cleavage in vitro [40]. Taken together, deletion or point mutation in *PARK7* results in the loss of function, which might give rise to disease development.

### 3.6. Conservation Analysis

**Figure 5:**
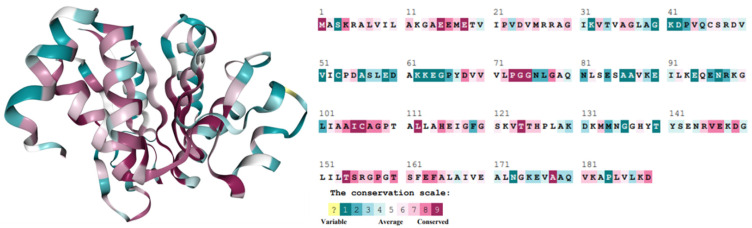
ConSurf plot showing residual conservation in the *PARK7* structure and sequence.

Conservation analysis of amino acids in a protein provides better insights into the residual evolution [42,43]. Here, in conservation analysis, the result showed that the amino acid stretches in *PARK7* ranges from 1–30, 67–75, 102–127, 145–169, 181–182, and 186–189 were highly conserved, while the stretches from amino acids 38–66, 76–101, 128–144, and 170–180 were less conserved. The analysis suggested that amino acids L166 and L172 are relatively conserved, and any mutations to these sites may result in the structural destabilization of *PARK7* (Figure 5).

### 3.7. Frustration Analysis

**Figure 6:**
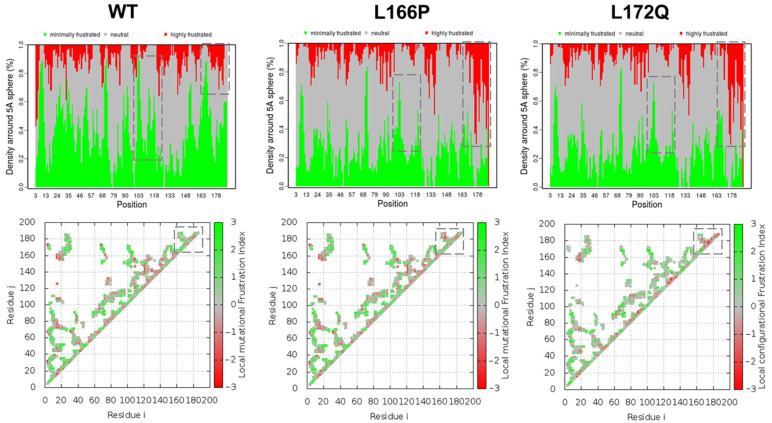
Frustration and configurational loss and gain contacts in WT, L166P, and L172Q, where minimally frustrated (green) and highly frustrated (red) residues are depicted. The upper and lower panels show the density and covariance matrices, respectively.

It is a well-established fact that the energy landscape of proteins is funneled toward the native ensemble, characterized by global minima [44]. Frustration analysis helps identify the frustration levels and their locations in protein structure, which can help understand how mutations can affect protein conformations and structural stability [44,45]. Towards this, the frustration energetics were investigated in *PARK7* and its mutants (L166P and L172Q). We comprehensively explored the local frustration ion indices in all three systems (Figure 6). The frustration indices showed that the C-terminus (residues 175–188) increased frustration (highlighted as dotted rectangles) when *PARK7* was mutated. However, the frustration was found to slightly decrease on the N-terminus in the first few residues spanning 1–5. Overall, the frustration indices suggested that mutations L166P and L172Q alter the *PARK7* frustration, which might be responsible for the instability of the protein.

## 4. Conclusions

SNPs are examined as the most successive hereditary variations related to various human diseases. Broad investigation of SNPs can offer understandings to comprehend the disease-causing component and help discover successful therapies for various complex diseases. In the current study, we examined various mutations in PARK7. The sequence-based and structural-based approaches showed that out of 152 mutations in the *PARK7* protein, 76 are considered destabilizing and deleterious. Out of these 76 mutations, 25 were found to be pathogenic. Aggregation propensity was carried out to examine 21 mutations of reduced stability and found that 9 pathogenic mutants accumulate and become insoluble. The structural alteration that occurred by the gain or loss of noncovalent interatomic interactions significantly impacts the amino acid. It may be mutated and become pathogenic, as shown by the extensive structural analysis approach. This study presents a thorough understanding of the pathogenic mutations and their possible effect on disease progression. After an extensive literature survey and analysis, two mutations (L166P & L172Q) were selected and explored in detail. The study suggested that L166P and L172Q mutations can alter the structure and function of PARK7, which might be responsible for the disease’s progression. The detailed understanding of *PARK7* mutations will help make therapeutic strategies for associated diseases, including Parkinson’s disease.

## Figures and Tables

**Table 1:** Identification of disease phenotype by using Rhapsody and PhD-SNP of high confidence missense variants of *PARK7* protein.

S. No.	Mutation ID	RHAPSODY		PhD-SNP
		*Score*	*Remarks*	
**1.**	K4R	0.466	Neutral	Neutral
**2.**	A6S	0.848	Deleterious	Neutral
**3.**	A6V	0.383	Neutral	Neutral
**4.**	L10M	0.819	Deleterious	Neutral
**5.**	L10P	0.917	Deleterious	Disease
**6.**	G13E	0.840	Deleterious	Disease
**7.**	E16G	0.897	Deleterious	Disease
**8.**	M17V	0.754	Deleterious	Neutral
**9.**	E18K	0.915	Deleterious	Disease
**10.**	V20M	0.429	Neutral	Neutral
**11.**	R27K	0.645	Neutral	Neutral
**12.**	A29V	0.733	Prob.Delet.	Disease
**13.**	T34I	0.411	Neutral	Neutral
**14.**	A36E	0.936	Deleterious	Neutral
**15.**	V44A	0.421	Neutral	Neutral
**16.**	C46R	0.865	Deleterious	Neutral
**17.**	S47G	0.598	Deleterious	Neutral
**18.**	R48G	0.671	Neutral	Neutral
**19.**	R48C	0.499	Pro. Neutral	Neutral
**20.**	R48H	0.410	Neutral	Neutral
**21.**	V51G	0.646	Neutral	Neutral
**22.**	C53F	0.684	Prob.Neutral	Neutral
**23.**	C53W	0.588	Deleterious	Neutral
**24.**	P54S	0.660	Neutral	Disease
**25.**	P54H	0.811	Deleterious	Disease
**26.**	S57I	0.621	Neutral	Neutral
**27.**	A61T	0.585	Neutral	Neutral
**28.**	A61E	0.749	Deleterious	Neutral
**29.**	D68H	0.885	Deleterious	Disease
**30.**	D68G	0.857	Deleterious	Disease
**31.**	D68V	0.903	Deleterious	Disease
**32.**	V70M	0.872	Deleterious	Neutral
**33.**	G75S	0.901	Deleterious	Disease
**34.**	G78S	0.797	Deleterious	Disease
**35.**	V88M	0.824	Deleterious	Neutral
**36.**	R98W	0.463	Neutral	Neutral
**37.**	G100D	0.677	Prob.Neutral	Disease
**38.**	G100V	0.343	Neutral	Neutral
**39.**	A104T	0.953	Deleterious	Disease
**40.**	A104S	0.812	Deleterious	Disease
**41.**	A107T	0.934	Deleterious	Neutral
**42.**	A107P	0.951	Deleterious	Disease
**43.**	A107S	0.923	Deleterious	Neutral
**44.**	L112P	0.949	Deleterious	Disease
**45.**	H115D	0.765	Deleterious	Neutral
**46.**	H115R	0.735	Prob.Delet.	Neutral
**47.**	T124R	0.913	Deleterious	Disease
**48.**	P127A	0.777	Deleterious	Disease
**49.**	P127S	0.798	Deleterious	Disease
**50.**	P127R	0.831	Deleterious	Disease
**51.**	K130T	0.627	Neutral	Neutral
**52.**	R145S	0.363	Neutral	Neutral
**53.**	R145C	0.838	Deleterious	Neutral
**54.**	R145P	0.493	Neutral	Disease
**55.**	V146M	0.826	Deleterious	Neutral
**56.**	V146G	0.879	Deleterious	Disease
**57.**	D149A	0.482	Prob. Neutral	Disease
**58.**	G150S	0.316	Neutral	Disease
**59.**	G150D	0.465	Neutral	Neutral
**60.**	T154A	0.939	Deleterious	Disease
**61.**	T154R	0.962	Deleterious	Disease
**62.**	R156W	0.581	Deleterious	Neutral
**63.**	G157A	0.89	Deleterious	Neutral
**64.**	P158S	0.783	Deleterious	Disease
**65.**	T160S	0.903	Deleterious	Neutral
**66.**	F164L	0.885	Deleterious	Neutral
**67.**	A165V	0.644	Deleterious	Disease
**68.**	L166P	0.915	Deleterious	Disease
**69.**	L172Q	0.85	Deleterious	Disease

**Table 2:** RSA, residue depth, and OSP scores of *PARK7* mutants predicted through the SDM2 server.

S. No.	Mutation	WT_RSA (%)	WT_DEPTH (Å)	WT_OSP	MT_RSA (%)	MT_DEPTH (Å)	MT_OSP	Outcome
**1.**	L10P	0	7.3	0.45	3.3	8.1	0.44	Reduced stability
**2.**	G13E	19.9	5.2	0.49	19.8	4.1	0.48	Reduced stability
**3.**	E16G	9.3	4.6	0.52	20.8	4.4	0.39	Reduced stability
**4.**	E18K	0	6.2	0.58	0.3	6.3	0.59	Reduced stability
**5.**	P54H	0	5.6	0.41	0.6	6.2	0.51	Reduced stability
**6.**	D68H	38.4	3.8	0.36	48.4	3.8	0.31	Reduced stability
**7.**	D68G	38.4	3.8	0.36	55	4	0.32	Reduced stability
**8.**	D68V	38.4	3.8	0.36	42.5	3.6	0.35	Reduced stability
**9.**	G75S	18.2	4.8	0.43	15.5	4.4	0.47	Reduced stability
**10.**	G78S	2	6.4	0.61	0.7	7.7	0.65	Reduced stability
**11.**	A104T	0	12.6	0.60	0.1	12.3	0.62	Reduced stability
**12.**	A104S	0	12.6	0.60	0	12.5	0.6	Reduced stability
**13.**	A107P	7.5	5	0.45	7.2	4.9	0.53	Reduced stability
**14.**	L112P	0.3	9.8	0.53	4.5	9.7	0.55	Reduced stability
**15.**	T124R	0	7.6	0.62	5.9	6.4	0.68	Reduced stability
**16.**	P127A	51.6	3.4	0.30	71.2	3.1	0.25	Increased stability
**17.**	P127S	51.6	3.4	0.30	69.8	3.3	0.24	Increased stability
**18.**	P127R	51.6	3.4	0.30	85.3	3.3	0.14	Increased stability
**19.**	V146G	18.2	3.7	0.39	33.5	3.8	0.27	Reduced stability
**20.**	T154A	0	9.7	0.54	0.5	9.8	0.50	Reduced stability
**21.**	T154R	0	9.7	0.54	0.3	10.4	0.66	Reduced stability
**22.**	P158S	32.3	3.5	0.34	34.6	3.5	0.31	Increased stability
**23.**	A165V	0	8.4	0.56	0	8.7	0.68	Reduced stability
**24.**	L166P	12.5	4.6	0.45	12.9	5	0.44	Reduced stability
**25.**	L172Q	4.6	5.1	0.45	5.4	4.9	0.40	Reduced stability

**Table 3:** Aggregation propensity prediction of *PARK7* mutants with the help of SODA.

S. No.	Mutation	SODA	Remarks
**1.**	L10P	24.49	More soluble
**2.**	G13E	3.11	More soluble
**3.**	E16G	−1.87	Less soluble
**4.**	E18K	−1.19	Less soluble
**5.**	P54H	−21.34	Less soluble
**6.**	D68H	−22.29	Less soluble
**7.**	D68G	−1.92	Less soluble
**8.**	D68V	−107.76	Less soluble
**9.**	G75S	−0.32	Less soluble
**10.**	G78S	0.17	More soluble
**11.**	A104T	−3.29	Less soluble
**12.**	A104S	4.20	More soluble
**13.**	A107P	3.90	More soluble
**14.**	L112P	7.28	More soluble
**15.**	T124R	4.75	More soluble
**16.**	V146G	7.045	More soluble
**17.**	T154A	1.59	More soluble
**18.**	T154R	5.65	More soluble
**19.**	A165V	−49.98	Less soluble
**20.**	L166P	18.93	More soluble
**21.**	L172Q	3.71	More soluble

**Table 4:** Prediction of noncovalent bonds of mutants and wild-type *PARK7* protein.

S. No.	Variant	van der Waals Interaction	Hydrogen Bonds	Ionic Interactions	Aromatic Contacts	Hydrophobic Contacts
**1.**	L10P	118	182	12	13	350
**2.**	G13E	110	182	12	13	347
**3.**	E16G	107	178	12	13	340
**4.**	E18K	108	180	12	13	353
**5.**	P54H	110	181	12	13	346
**6.**	D68H	110	180	12	13	347
**7.**	D68G	108	180	12	13	342
**8.**	D68V	110	180	12	13	356
**9.**	G75S	133	210	12	13	422
**10.**	G78S	132	209	12	13	422
**11.**	A104T	110	181	12	13	346
**12.**	A104S	108	181	12	13	341
**13.**	A107P	130	210	12	13	423
**14.**	L112P	112	180	12	13	341
**15.**	T124R	114	186	13	13	349
**16.**	V146G	116	183	12	13	358
**17.**	T154A	109	180	12	13	343
**18.**	T154R	111	180	12	13	349
**19.**	A165V	111	181	12	13	357
**20.**	L166P	111	179	12	13	339
**21.**	L172Q	110	182	12	13	334
**22.**	Wild-type	114	181	12	13	347

## Data Availability

Not applicable.

## Supplementary Materials

The following are available online at https://www.mdpi.com/article/10.3390/jpm12020220/s1. Table S1: Sequence-based analysis of 152 mutations of *PARK7* protein, Table S2: Structure-based analysis of 76 mutations of *PARK7* protein.
