# Supplementary material for: Impact of Single Amino Acid Substitutions in Parkinsonism-Associated Deglycase-PARK7 and Their Association with Parkinson’s Disease
Source: J Pers Med. 2022 Feb 5;12(2):220. doi: 10.3390/jpm12020220 (PMC8878504; doi:10.3390/jpm12020220)
Supplement: Supplementary file 1 [file jpm-12-00220-s001.zip › jpm-1518127-supplementary.pdf]

Supplementary Tables

Impact of single amino acid substitutions in parkinsonism associated deglycase-PARK7 and their association with Parkinson's disease

Farah Anjum[1], Namrata Joshia[2], Taj Mohammad[3], Alaa Shafie[1], Fahad A. Alhumaydhi[4], Mohammad A. Aljasir[4], Moyad J.S. Shahwan[5], Bekhzod Abdullaev[6], Mohd Adnan[7], Abdelbaset Mohamed Elasbali[8], Visweswara Rao Pasupuleti[9,10,11,*], Md Imtaiyaz Hassan[3,*]

[1]*Department of Clinical Laboratory Sciences, College of Applied Medical Sciences, Taif University, P.O. Box 11099, Taif 21944, Saudi Arabia. f2016anjum@gmail.com (F.A.); dr.alaa.shafie.tu@gmail.com (A.S.)*

[2]*Department of Computer Science, Jamia Millia Islamia, Jamia Nagar, New Delhi 110025, INDIA. namrata8750451546@gmail.com (N.J.)*

[3]*Centre for Interdisciplinary Research in Basic Sciences, Jamia Millia Islamia, Jamia Nagar, New Delhi 110025, INDIA. taj144796@st.jmi.ac.in (T.M.)*

[4]*Department of Medical Laboratories, College of Applied Medical Sciences, Qassim University, Buraydah, Saudi Arabia. f.alhumaydhi@qu.edu.sa (F.A.A.); Mjasr@qu.edu.sa (M.A.A.)*

[5]*College of Pharmacy & Health sciences, Ajman University, Ajman, UAE. m.shahwan@ajman.ac.ae (M.J.S.S.)*
[6]*Scientific Department, Akfa University, Tashkent, Uzbekistan. b.abdullaev@akfauniversity.org (B.A.)*

[7]*Department of Biology, College of Science, University of Hail, Hail, P O Box 2440, Saudi Arabia. drmohdadnan@gmail.com (M.A.)*

[8]Clinical Laboratory Science, College of Applied Sciences-Qurayyat, Jouf University, Saudi Arabia.

aeelasbali@ju.edu.sa (A.M.E.)

[9]*Department of Biomedical Sciences and Therapeutics, Faculty of Medicine & Health Sciences, Uni-versiti Malaysia Sabah, Kota Kinabalu, 44800, Sabah, Malaysia.*

[10]*Department of Biochemistry, Faculty of Medicine and Health Sciences, Abdurrab University, Pek-anbaru, Riau, Indonesia.*

[11]*Centre for Excellence in Biomaterials Engineering (CoEBE), AIMST University, 08100 Bedong, Kedah, Malaysia.*

**Correspondence: pvrao@ums.edu.my (V.R.P.); mihassan@jmi.ac.in (M.I.H.)*

Table S1: Sequence-based analysis of 152 mutations of PARK7 protein.

S. No.	Mutation Id	Mutation Assessor	SIFT	PROVEAN	PON-2	PolyPhen-2
1.	K4E	High	Damaging	Deleterious	Unknown	Probably Damaging
2.	K4R	Medium	Damaging	Neutral	Pathogenic	Possibly Damaging
3.	A6S	Medium	Damaging	Deleterious	Unknown	Probably Damaging
4.	A6V	Low	Damaging	Deleterious	Unknown	Probably Damaging
5.	L10M	Medium	Damaging	Neutral	Unknown	Probably Damaging
6.	L10V	Medium	Tolerated	Neutral	Unknown	Possibly Damaging
7.	L10P	High	Damaging	Deleterious	Pathogenic	Probably Damaging
8.	K12E	Neutral	Tolerated	Neutral	Unknown	Benign
9.	G13E	High	Damaging	Deleterious	Pathogenic	Probably Damaging
10.	A14T	Low	Tolerated	Deleterious	Pathogenic	Benign
11.	E16G	High	Damaging	Deleterious	Unknown	Probably Damaging
12.	M17L	Medium	Tolerated	Deleterious	Unknown	Benign
13.	M17V	Low	Damaging	Deleterious	Unknown	Possibly Damaging
14.	M17I	Low	Tolerated	Deleterious	Unknown	Benign
15.	E18K	High	Damaging	Deleterious	Unknown	Probably Damaging
16.	E18D	High	Damaging	Deleterious	Unknown	Probably Damaging
17.	T19M	Medium	Tolerated	Neutral	Pathogenic	Probably Damaging
18.	V20A	Medium	Damaging	Deleterious	Pathogenic	Benign
19.	V23I	Neutral	Tolerated	Neutral	Unknown	Benign
20.	V25I	Low	Tolerated	Neutral	Unknown	Benign
21.	M26L	Neutral	Tolerated	Neutral	Unknown	Benign
22.	M26I	Low	Damaging	Neutral	Unknown	Benign
23.	R27K	Medium	Damaging	Deleterious	Pathogenic	Possibly Damaging
24.	R28Q	High	Damaging	Deleterious	Pathogenic	Probably Damaging
25.	A29G	Low	Tolerated	Neutral	Unknown	Benign
26.	A29V	Medium	Damaging	Deleterious	Unknown	Probably Damaging
27.	T34I	Low	Damaging	Deleterious	Unknown	Possibly Damaging
28.	V35I	Low	Tolerated	Neutral	Unknown	Benign
29.	A36E	High	Damaging	Deleterious	Pathogenic	Probably Damaging
30.	A36V	Low	Damaging	Deleterious	Unknown	Probably Damaging
31.	A39T	Neutral	Tolerated	Neutral	Neutral	Benign
32.	A39S	Neutral	Tolerated	Neutral	Unknown	Benign
33.	D42A	Low	Tolerated	Deleterious	Unknown	Benign
34.	D42V	Low	Tolerated	Deleterious	Unknown	Benign
35.	D42E	Neutral	Tolerated	Neutral	Neutral	Benign
36.	V44A	Medium	Damaging	Deleterious	Pathogenic	Probably Damaging
37.	Q45H	Low	Tolerated	Neutral	Unknown	Benign
38.	C46R	Medium	Damaging	Deleterious	Pathogenic	Probably Damaging
39.	S47G	Medium	Damaging	Deleterious	Pathogenic	Probably Damaging
40.	R48G	Medium	Damaging	Deleterious	Pathogenic	Probably Damaging
41.	R48C	Medium	Damaging	Deleterious	Pathogenic	Probably Damaging
42.	R48H	Low	Damaging	Deleterious	Pathogenic	Probably Damaging
43.	V51G	Medium	Tolerated	Deleterious	Unknown	Benign

44.	I52V	Low	Tolerated	Neutral	Unknown	Benign
45.	C53F	Medium	Tolerated	Deleterious	Unknown	Possibly Damaging
46.	C53W	Medium	Damaging	Deleterious	Unknown	Probably Damaging
47.	P54S	Medium	Damaging	Deleterious	Pathogenic	Probably Damaging
48.	P54H	Medium	Damaging	Deleterious	Pathogenic	Benign
49.	A56T	Neutral	Tolerated	Neutral	Unknown	Benign
50.	S57T	Low	Tolerated	Neutral	Unknown	Benign
51.	S57I	Medium	Damaging	Deleterious	Pathogenic	Benign
52.	E59D	Neutral	Tolerated	Neutral	Unknown	Benign
53.	A61T	Medium	Damaging	Deleterious	Pathogenic	Possibly Damaging
54.	A61E	Medium	Damaging	Deleterious	Pathogenic	Probably Damaging
55.	A61V	Low	Tolerated	Neutral	Pathogenic	Benign
56.	K63R	Low	Tolerated	Neutral	Unknown	Benign
57.	P66Q	Low	Tolerated	Neutral	Pathogenic	Benign
58.	D68H	High	Damaging	Deleterious	Pathogenic	Probably Damaging
59.	D68G	High	Damaging	Deleterious	Unknown	Probably Damaging
60.	D68V	High	Damaging	Deleterious	Pathogenic	Probably Damaging
61.	V69M	Low	Damaging	Neutral	Pathogenic	Possibly Damaging
62.	V70M	Medium	Damaging	Deleterious	Unknown	Probably Damaging
63.	P73L	High	Damaging	Deleterious	Pathogenic	Probably Damaging
64.	G75S	High	Damaging	Deleterious	Pathogenic	Probably Damaging
65.	N76D	Medium	Tolerated	Neutral	Unknown	Benign
66.	L77M	Medium	Tolerated	Neutral	Unknown	Possibly Damaging
67.	L77R	Low	Tolerated	Neutral	Pathogenic	Benign
68.	G78S	Medium	Damaging	Deleterious	Pathogenic	Probably Damaging
69.	A79T	Low	Damaging	Neutral	Pathogenic	Probably Damaging
70.	A79S	Low	Tolerated	Neutral	Unknown	Possibly Damaging
71.	A79V	Medium	Damaging	Deleterious	Unknown	Probably Damaging
72.	N81S	Medium	Tolerated	Neutral	Unknown	Benign
73.	S83F	Medium	Damaging	Deleterious	Unknown	Probably Damaging
74.	A87V	Neutral	Tolerated	Neutral	Unknown	Benign
75.	V88M	Medium	Damaging	Deleterious	Unknown	Possibly Damaging
76.	I91T	Low	Damaging	Deleterious	Unknown	Possibly Damaging
77.	R98W	Medium	Damaging	Deleterious	Unknown	Benign
78.	R98Q	Neutral	Tolerated	Neutral	Unknown	Benign
79.	G100S	Low	Tolerated	Neutral	Unknown	Benign
80.	G100D	Medium	Damaging	Neutral	Pathogenic	Possibly Damaging
81.	G100A	Medium	Tolerated	Neutral	Unknown	Benign
82.	G100V	Medium	Damaging	Deleterious	Unknown	Possibly Damaging
83.	L101V	Medium	Tolerated	Neutral	Unknown	Benign
84.	A104T	High	Damaging	Deleterious	Pathogenic	Probably Damaging
85.	A104S	Medium	Damaging	Deleterious	Unknown	Possibly Damaging
86.	A107T	High	Damaging	Deleterious	Pathogenic	Probably Damaging
87.	A107P	High	Damaging	Deleterious	Pathogenic	Probably Damaging
88.	A107S	Medium	Damaging	Deleterious	Unknown	Probably Damaging
89.	T110A	Low	Damaging	Deleterious	Unknown	Benign

90.	T110S	Low	Damaging	Deleterious	Unknown	Possibly Damaging
91.	T110I	Low	Tolerated	Deleterious	Unknown	Possibly Damaging
92.	L112P	High	Damaging	Deleterious	Unknown	Probably Damaging
93.	A114V	Medium	Damaging	Deleterious	Unknown	Benign
94.	H115D	High	Damaging	Deleterious	Pathogenic	Probably Damaging
95.	H115R	Medium	Damaging	Deleterious	Pathogenic	Probably Damaging
96.	E116K	Neutral	Tolerated	Neutral	Unknown	Benign
97.	T124R	High	Damaging	Deleterious	Unknown	Probably Damaging
98.	T125I	Low	Damaging	Deleterious	Unknown	Probably Damaging
99.	P127A	Medium	Damaging	Deleterious	Unknown	Probably Damaging
100.	P127S	Medium	Damaging	Deleterious	Unknown	Probably Damaging
101.	P127R	High	Damaging	Deleterious	Pathogenic	Probably Damaging
102.	L128F	Low	Damaging	Neutral	Unknown	Benign
103.	L128P	Low	Tolerated	Neutral	Pathogenic	Benign
104.	K130T	Medium	Damaging	Deleterious	Pathogenic	Probably Damaging
105.	K132E	Low	Tolerated	Neutral	Unknown	Benign
106.	M133I	Neutral	Tolerated	Neutral	Unknown	Benign
107.	M134V	Neutral	Tolerated	Neutral	Unknown	Benign
108.	N135D	Neutral	Tolerated	Neutral	Unknown	Benign
109.	H138R	Medium	Tolerated	Neutral	Pathogenic	Possibly Damaging
110.	T140A	Low	Tolerated	Neutral	Unknown	Benign
111.	T140S	Low	Tolerated	Neutral	Neutral	Benign
112.	N144S	Neutral	Tolerated	Neutral	Unknown	Benign
113.	N144K	Neutral	Damaging	Neutral	Unknown	Benign
114.	R145S	Medium	Tolerated	Deleterious	Pathogenic	Possibly Damaging
115.	R145C	High	Damaging	Deleterious	Pathogenic	Probably Damaging
116.	R145H	High	Damaging	Deleterious	Pathogenic	Possibly Damaging
117.	R145P	Medium	Damaging	Deleterious	Pathogenic	Benign
118.	V146M	High	Damaging	Deleterious	Pathogenic	Probably Damaging
119.	V146G	High	Damaging	Deleterious	Unknown	Probably Damaging
120.	K148T	Low	Tolerated	Neutral	Unknown	Benign
121.	D149A	High	Damaging	Deleterious	Unknown	Probably Damaging
122.	G150S	Medium	Damaging	Deleterious	Pathogenic	Possibly Damaging
123.	G150D	Medium	Tolerated	Deleterious	Pathogenic	Benign
124.	T154A	High	Damaging	Deleterious	Pathogenic	Probably Damaging
125.	T154R	High	Damaging	Deleterious	Pathogenic	Probably Damaging
126.	R156W	High	Damaging	Deleterious	Pathogenic	Probably Damaging
127.	R156Q	Low	Tolerated	Deleterious	Pathogenic	Possibly Damaging
128.	G157R	High	Damaging	Deleterious	Pathogenic	Probably Damaging
129.	G157A	Medium	Damaging	Deleterious	Pathogenic	Probably Damaging
130.	P158S	Medium	Damaging	Deleterious	Pathogenic	Probably Damaging
131.	G159E	High	Damaging	Deleterious	Pathogenic	Probably Damaging
132.	T160S	Medium	Damaging	Deleterious	Unknown	Probably Damaging
133.	E163K	Medium	Tolerated	Deleterious	Unknown	Benign
134.	F164L	Medium	Damaging	Deleterious	Pathogenic	Probably Damaging
135.	A165V	Medium	Damaging	Deleterious	Unknown	Probably Damaging

136.	L166P	High	Damaging	Deleterious	Pathogenic	Probably Damaging
137.	A167T	Low	Tolerated	Neutral	Neutral	Benign
138.	A167G	Medium	Tolerated	Neutral	Neutral	Possibly Damaging
139.	A167V	Medium	Tolerated	Neutral	Unknown	Benign
140.	I168V	Medium	Tolerated	Neutral	Unknown	Possibly Damaging
141.	A171S	Low	Tolerated	Neutral	Neutral	Benign
142.	L172Q	High	Damaging	Deleterious	Pathogenic	Probably Damaging
143.	K175E	Low	Tolerated	Neutral	Unknown	Benign
144.	E176A	Low	Tolerated	Deleterious	Unknown	Benign
145.	A178V	Low	Tolerated	Neutral	Unknown	Benign
146.	A179V	Low	Tolerated	Neutral	Neutral	Benign
147.	Q180E	Neutral	Tolerated	Neutral	Unknown	Benign
148.	V181M	Medium	Damaging	Neutral	Unknown	Probably Damaging
149.	V181L	Low	Tolerated	Neutral	Unknown	Benign
150.	L185F	Medium	Damaging	Neutral	Unknown	Possibly Damaging
151.	K188E	Medium	Tolerated	Neutral	Unknown	Benign
152.	D189G	Low	Damaging	Neutral	Unknown	Possibly Damaging

Table S2: Structure-based analysis of 76 mutations of PARK7 protein.

S. No.	Mutation	SDM2	mCSM	Mupro
		**Outcome**	**Outcome**	**Outcome**
1.	K4E	Increase	Increase	Decrease
2.	K4R	Increase	Decrease	Decrease
3.	A6S	Decrease	Decrease	Decrease
4.	A6V	Increase	Decrease	Decrease
5.	L10M	Decrease	Decrease	Decrease
6.	L10P	Decrease	Decrease	Decrease
7.	G13E	Decrease	Decrease	Decrease
8.	E16G	Decrease	Decrease	Decrease
9.	M17V	Decrease	Decrease	Decrease
10.	E18K	Decrease	Decrease	Decrease
11.	E18D	Decrease	Decrease	Decrease
12.	T19M	Increase	Decrease	Decrease
13.	V20A	Increase	Decrease	Decrease
14.	R27K	Decrease	Decrease	Decrease
15.	R28Q	Increase	Decrease	Decrease
16.	A29V	Decrease	Decrease	Decrease
17.	T34I	Increase	Decrease	Decrease
18.	A36E	Decrease	Decrease	Decrease
19.	A36V	Increase	Decrease	Increase
20.	V44A	Decrease	Decrease	Decrease
21.	C46R	Decrease	Decrease	Decrease
22.	S47G	Increase	Decrease	Decrease
23.	R48G	Decrease	Decrease	Decrease
24.	R48C	Increase	Decrease	Decrease
25.	R48H	Increase	Decrease	Decrease

26.	C53F	Decrease	Decrease	Decrease
27.	C53W	Increase	Decrease	Decrease
28.	P54S	Decrease	Decrease	Decrease
29.	P54H	Decrease	Decrease	Decrease
30.	S57I	Decrease	Decrease	Decrease
31.	A61T	Decrease	Decrease	Decrease
32.	A61E	Decrease	Decrease	Decrease
33.	D68H	Decrease	Decrease	Decrease
34.	D68G	Decrease	Decrease	Decrease
35.	D68V	Decrease	Increase	Decrease
36.	V70M	Decrease	Decrease	Decrease
37.	P73L	Increase	Decrease	Increase
38.	G75S	Decrease	Decrease	Decrease
39.	G78S	Decrease	Decrease	Decrease
40.	V88M	Decrease	Decrease	Decrease
41.	R98W	Decrease	Decrease	Decrease
42.	G100D	Decrease	Decrease	Decrease
43.	G100V	Increase	Decrease	Decrease
44.	A104T	Decrease	Decrease	Decrease
45.	A104S	Decrease	Decrease	Decrease
46.	A107T	Decrease	Decrease	Decrease
47.	A107P	Decrease	Decrease	Decrease
48.	A107S	Decrease	Decrease	Decrease
49.	L112P	Decrease	Decrease	Decrease
50.	H115D	Decrease	Decrease	Decrease
51.	H115R	Decrease	Decrease	Decrease
52.	T124R	Decrease	Decrease	Decrease
53.	P127A	Increase	Decrease	Decrease
54.	P127S	Increase	Decrease	Decrease
55.	P127R	Increase	Decrease	Decrease
56.	K130T	Decrease	Decrease	Decrease
57.	R145S	Decrease	Decrease	Decrease
58.	R145C	Decrease	Decrease	Decrease
59.	R145H	Increase	Decrease	Decrease
60.	R145P	Decrease	Decrease	Decrease
61.	V146M	Decrease	Decrease	Decrease
62.	V146G	Decrease	Decrease	Decrease
63.	D149A	Increase	Decrease	Decrease
64.	G150S	Decrease	Decrease	Decrease
65.	G150D	Decrease	Decrease	Decrease
66.	T154A	Decrease	Decrease	Decrease
67.	T154R	Decrease	Decrease	Decrease
68.	R156W	Increase	Decrease	Decrease
69.	G157A	Decrease	Decrease	Decrease
70.	P158S	Increase	Decrease	Decrease
71.	G159E	Increase	Decrease	Increase
72.	T160S	Decrease	Decrease	Decrease
73.	F164L	Decrease	Decrease	Decrease
74.	A165V	Decrease	Increase	Decrease

75.	L166P	Decrease	Decrease	Decrease
76.	L172Q	Decrease	Decrease	Decrease
